# Teleradiology: The Indian perspective

**DOI:** 10.4103/0971-3026.45337

**Published:** 2009-02

**Authors:** Nishigandha Burute, Bhavin Jankharia

**Affiliations:** Radiologists, Piramal Diagnostics - Jankharia Imaging, Mumbai 400 004, India

## Introduction

The demand for diagnostic and image interpretation services in radiology is growing rapidly all over the world. This has highlighted two issues: the lack of adequate staff for providing interpretative coverage and the lack of specialty expertise. To some extent, these problems can be overcome by utilizing robust communication and image transfer systems to draw on the expertise of distantly located radiologists. This process whereby images are transferred to distant locations for the purpose of interpretation and diagnosis is termed teleradiology.

## Telemedicine

Telemedicine is a broad term encompassing all methods where the doctor-patient interaction is not on-site and some form of telecommunication is used. Also called telehealth, online health, or e-health, telemedicine has made long strides since its inception.

## Teleradiology

Teleradiology is a branch of telemedicine in which telecommunication systems are used to transmit radiological images from one location to another. Interpretation of all noninvasive imaging studies, such as digitized x-rays, CT, MRI, ultrasound, and nuclear medicine studies, can be carried out in such a manner.

The earliest efforts in teleradiology probably date back to 1929, when dental x-rays were transmitted with the help of telegraph to a distant location.[1] An early attempt at using the Web in an emergency medical situation describes the use of digital cameras to take clinical photographs and scanners to scan radiographs, conversion of the resulting digital images to a JPEG format using Adobe Photoshop, and then transmission via the Internet.

Today, digitized images are transmitted around the globe via high-speed telecommunication links on a regular basis.

## Need for teleradiology

Teleradiology took birth partly due to the imbalance between the demand and availability of diagnostic services. The high demand for radiology services in places such as USA, UK, and Singapore often could not be fulfilled by the number of available in-house healthcare professionals. In the USA, while the number of scans being performed has been increasing, there is a persisting shortage of radiologists. In Singapore, there is a paucity of radiologists for night coverage. In the UK, a radiologist takes 21 days on average to submit an MRI report.

The lack of availability of timely diagnostic services causes great problems for clinicians during emergencies and during the night hours. Moreover, the Health Care Financing Administration (HCFA) in the US mandates round-the-clock services in every hospital. By outsourcing radiology reporting to places such as Australia, Europe, and some Asian countries (including India) hospitals in the USA, UK, and Singapore can be assured of competent and timely professional help. The immediate availability of diagnostic services, which is extremely important during medical emergencies, is a big advantage that outsourcing offers. Outsourcing of ‘on-call’ night reporting is popularly called ‘nighthawking.’

Another reason for the growth of teleradiology is that most parts of rural India do not have good radiological services and personnel. With teleradiology, this deficiency can be overcome by using the help of more experienced personnel in the larger centers in the cities. Also, even in the cities, not all imaging centers have subspecialty expertise; difficult cases in specific areas of radiology can be sent to experts for their opinion.

## Acquisition, transfer, and viewing of images

Images need to be acquired, stored, transferred, and viewed.

### Acquisition of images

Today, virtually all radiology equipment is fully DICOM compliant. Thus, images can be stored on a network or a workstation in the DICOM format. Lossy and lossless compression is possible; varying degrees of loss of information may be acceptable, depending upon the modality and the clinical situation. Plain radiographs obtained non-digitally may need to be scanned. Currently, mammography images remain the last barrier to reliable teleradiology; this is due to the large file sizes and issues related to the image resolution required to detect microcalcifications.

### Transfer of images

In the early days, transfer of images was performed over telephone lines using modems, sometimes with speeds as low as 2400 bps. Today, high-speed lines are available, allowing different centers to connect directly or over the Internet for transmission of images. Images may be directly transferred or streamed, depending upon the software being used.

### Viewing of images

Image viewing requires a workstation that can display high-resolution images. Many types of software are currently available, e.g., EFilm, which allows viewing, manipulation, measurements, 3D reconstructions, etc.

## Conveying reports

With the advent of PACS (picture archiving and communication system) RIS (radiology information system) is now integrated into the teleradiology system, resulting in efficient and instant communication of findings to the clinician or surgeon.

## History of teleradiology in India

The practice of teleradiology in India dates back to not more than a decade. The first successful use of teleradiology in India was in 1996 by a private-sector imaging center called Jankharia Imaging in Mumbai. A simple system for transferring images from the imaging center to the homes of the individual doctors was set up, primarily, to report emergency CT scans. The first public demonstration of teleradiology in practice was made by Siemens at the Annual Congress of the Indian Radiology and Imaging Association (IRIA) in 1997, where they demonstrated the transfer of radiological images from a Siemens AR.C scanner to the conference site. Subsequently, Wipro GE demonstrated teleradiology capabilities for their entire range of scanners.

The first teleradiology company in India, Teleradiology Solutions, was set up in 2002 with its base in Bangalore. Dr Arjun Kalyanpur and his colleagues, all US board-certified radiologists, read scans for hospitals in the USA; these services were offered for places in Singapore and India as well. Wipro Technologies has been an early mover in providing nighthawk and 3D reconstruction services. Many companies have announced their intentions to enter this market and some small enterprises provide services for preliminary reads.

## Current situation and issues

### International outsourcing

India has distinct advantages when it comes to teleradiology; for example:

1. *Cost:* An MRI in India, performed on a state-of-the-art scanner, costs Rs. 6000 (approx. 150 USD). The professional fee component is usually 10-15%, i.e., 15–25 USD. At these rates, having an Indian radiologist report outsourced scans can offer a significant monetary advantage.
2. *Cheap labor:* The salary of an Indian radiologist working in the field of CT and MRI, 5 years post-MD (Indian board certification), would usually be close to or less than Rs. 2 lakhs per month (i.e., approximately 5000 USD per month or 60,000 USD per year)[2]; in contrast, a comparably qualified radiologist in the USA would be earning approximately 350,000 USD per year.[3]
3. *Time difference:* The time difference between the USA and India is a distinct advantage, especially for nighthawk services. When it is night in the USA, it is daytime in India; this means that it would be possible for an Indian radiologist, working during the daytime, to interpret images with better quality and a greater accuracy than would the US radiologist in his night shift hours.
4. *Skilled support staff:* India also has a distinct advantage in the form of high-caliber information technology (IT) and business process outsourcing (BPO) manpower, as also a great number of engineers trained in the basic skills required for off shore jobs.

Ideally, these factors should have led to a significant growth of the teleradiology industry in India. However, the reality is that except for one company, i.e., Teleradiology Solutions, there is no other company practicing teleradiology to any significant extent in the country. There are some enterprises doing preliminary ‘ghost reporting’ for facilities in the USA, but this work is either in ethical grey zones, if not actually illegal, as far as the USA is concerned though not illegal from the Indian perspective. A couple of companies, including Wipro Technologies and the Manipal Group of Hospitals, are now providing teleradiology support for 3D reconstructions, thus speeding up the work for radiologists and technologists in the USA.. This work does not require board-certification since there is no actual interpretation being done.

The main hurdles for Indian teleradiology are as follows:

1. Lack of board-certified radiologists in India: A radiologist who goes to the US for board certification may be reluctant to return to India in view of the large differences in salaries and compensations. Those few who do come back and practice teleradiology may do so for lifestyle, “Indian” values, or family reasons.
2. ‘Third-world’ status and credibility: Despite the growth of medical facilities in India and the reasonably high levels of quality, people in the West are still wary of having Indian radiologists in India interpret studies.
3. Political concerns: The media hype on outsourcing to India, which is true for many fields, has had an impact on teleradiology outsourcing. In reality, however, there has not been much outsourcing of radiology services to India and the growth of this industry remains pretty dismal.
4. Small markets: The US, UK, and Singapore[4] are the main markets now open for Indian teleradiology. The countries that are likely to seek teleradiology outsourcing would be English-speaking countries and the ones where the cost differential makes economic sense. Not too many countries fulfill these criteria and in fact some European countries as well as Australia[5] and New Zealand[6] have been successful in leveraging their advantages to become hubs for outsourcing.

All these points are also issues with respect to the UK, more so because of how politically sensitive all heath-care issues in the UK are.

## National teleradiology

The main issues affecting the growth of teleradiology within the country are the following:

1. *Cost:* Radiology studies in this country are priced low, and centers can find it difficult to afford the services of teleradiologists.
2. *Quality issues:* Many rural and semiurban centers do not have qualified radiologists but they do not mind the lack of quality reporting and often leave it to the referring clinicians to read the images themselves.

Currently, the use of teleradiology within the country is confined to practices with multiple centers transferring images to each other or to a central hub. Rural-urban or generalists-to-specialists transfers are not yet very popular, mainly due to cost constraints. The earlier barriers related to the nonavailability of adequate bandwidth no longer exist in most parts of the country due to the rapid growth of telecommunication provider companies. The need of teleradiology though is considerable. Many district hospitals have CT scanners, but qualified and competent radiologists are few and far between. Most such hospitals are government and municipal facilities, and it has been difficult to get them to go in for teleradiology solutions from private practitioners. This is despite the request by ex-President Dr Abdul Kalam, who advised those concerned to take advantage of the benefits of telemedicine for helping the needy in the rural areas in a cost-effective manner.[7]

## Other potential applications and business opportunities

### Research

Many research groups or clinical research outsourcing (CRO) practices need radiology images to be reported, e.g., measurement of the size of tumors in oncology trials. Here there are very few entry barriers such as the need for board certification, etc., and there is significant scope for teleradiology outsourcing in this area.

### Teaching

Using multiple Web-based applications that allow real-time display of presentations, lectures can be now taken for audiences across the globe.

## Conclusion

Teleradiology has potential as a business model. However, numerous logistical issues have prevented its growth in India.

## References

[1] (1929). Sending dental X-rays by telegraph. Dent Radiogr Photogr.

[2] Levy F, Yu KH (2006). Offshoring radiology services to India. IPC.

[3] (2004). Some U.S. hospitals outsourcing work. Shortage of radiologists spurs growing telemedicine trend MSNBC news.

[4] Levy F, Yu KZH (2006). Offshoring radiology services to India. IPC.

[5] Crowe BL (2001). A review of the experience with teleradiology in Australia. J Telemed Telecare.

[6] Lawrence S (2006). Lau clinical teleradiology in Australia: Practice and standards. J Am Coll Radiol.

[7] (2007). Kalam Urges for Using Tele-radiology to Help Rural Poor. News on IT in Healthcare. MedIndia.

